# Supplementary material for: Adaptation and serial choice bias for low-level visual features are unaltered in autistic adolescents
Source: J Vis. 2022 May 3;22(6):1. doi: 10.1167/jov.22.6.1 (PMC9078051; doi:10.1167/jov.22.6.1)
Supplement: Supplement 1 [file jovi-22-6-1_s001.docx]

**Supplemental materials**

Supplemental table 1: Autism Diagnostic Interview – Revised

| **ASD group member** | **Scales** | | |
| --- | --- | --- | --- |
|  | Social interaction | Communication and language | Restricted and repetitive behaviors |
| 1 | 12 | 12 | 3 |
| 2 | 19 | 18 | 5 |
| 3 | 14 | 15 | 2 |
| 4 | 7 | 18 | 1 |
| 5 | 18 | 18 | 2 |
| 6 | 10 | 9 | 2 |
| 7 | 9 | 11 | 1 |
| 8 | 14 | 7 | 0 |
| 9 | 26 | 21 | 3 |
| 10 | 20 | 17 | 2 |
| 11 | 11 | 8 | 3 |
| 12 | 10 | 12 | 4 |
| 13 | 19 | 10 | 5 |
| 14 | 6 | 14 | 2 |
| 15 | 13 | 4 | 4 |
| 16 | 19 | 17 | 10 |
| 17 | 29 | 19 | 4 |
| 18 | 25 | 18 | 4 |
| 19 | 24 | 12 | 4 |
| 20 | 30 | 17 | 9 |
| 21 | 31 | 21 | 6 |
| 22 | 8 | 13 | 8 |
| 23 | 10 | 11 | 2 |
| 24 | 15 | 15 | 0 |
| 25 | 37 | 20 | 9 |
| 26 | 19 | 13 | 5 |
| 27 | 28 | 17 | 1 |
| 28 | 9 | 12 | 2 |
| 29 | 18 | 14 | 2 |
| 30 | 32 | 17 | 17 |
| 31 | 18 | 9 | 6 |

Supplemental table 2: GLMM fixed factors for a model over all participants (N = 64) that predicts the current decision based on current- and previous trial factors, group (ASD vs TD), and IQ.

|  | Estimate (*b*) | *SE* | *z* | *p* |
| --- | --- | --- | --- | --- |
| Single factors |  |  |  |  |
| *Intercept* | 0.1744 | 0.0491 | 3.553 | **3.81e-4** |
| *group* | -0.0625 | 0.0520 | -1.201 | 0.230 |
| *cStimIdent* | 1.4877 | 0.0759 | 19.611 | **< 2e-16** |
| *pDecision* | 0.0723 | 0.0329 | 2.201 | **0.028** |
| *pStimIdent* | -0.0153 | 0.0345 | -0.444 | 0.657 |
| *pRt* | -0.0019 | 0.0149 | -0.126 | 0.900 |
| *TIQ* | 0.0572 | 0.0525 | 1.089 | 0.276 |
| Interactions with *pRt* |  |  |  |  |
| *pDecision x pRt* | -0.0627 | 0.0156 | -4.012 | **6.02e-5** |
| *pStimIdent x pRt* | 0.0066 | 0.0228 | 0.288 | 0.773 |
| Interactions with *group* (and *pRt*) |  |  |  |  |
| *cStimIdent x group* | -0.0366 | 0.0802 | -0.456 | 0.648 |
| *pDecision x group* | 0.0487 | 0.0347 | 1.404 | 0.160 |
| *pStimIdent x group* | 0.0308 | 0.0364 | 0.845 | 0.398 |
| *pRt x group* | -0.0020 | 0.0162 | -0.125 | 0.901 |
| *pDecision x pRt x group* | -0.0308 | 0.0171 | -1.801 | 0.072 |
| *pStimIdent x pRt x group* | -0.0240 | 0.0250 | -0.958 | 0.338 |
| Interactions with *TIQ* (and *pRt*) |  |  |  |  |
| *cStimIdent x TIQ* | 0.2535 | 0.0809 | 3.132 | **1.73e-3** |
| *pDecision x TIQ* | 0.0435 | 0.0354 | 1.229 | 0.219 |
| *pStimIdent x TIQ* | -0.0450 | 0.0374 | -1.201 | 0.230 |
| *pRt x TIQ* | -0.0196 | 0.0156 | -1.255 | 0.210 |
| *pDecision x pRt x TIQ* | -0.0264 | 0.0164 | -1.612 | 0.107 |
| *pStimIdent x pRt x TIQ* | -0.0232 | 0.0240 | -0.966 | 0.334 |
| Button mapping |  |  |  |  |
| *cButtonMapping* | 0.0674 | 0.0186 | 3.628 | **2.86e-4** |
| *cButtonMapping x group* | 0.0130 | 0.0197 | 0.661 | 0.509 |
| *cButtonMapping x TIQ* | -0.0123 | 0.0199 | -0.614 | 0.539 |
| *pButtonXcButtonMapping* | -0.0773 | 0.0215 | -3.586 | **3.36e-4** |
| *pButtonXcButtonMapping x group* | -0.0014 | 0.0228 | -0.061 | 0.951 |
| *pButtonXcButtonMapping x TIQ* | 0.0487 | 0.0231 | 2.110 | **0.035** |
